# Transcriptomic Responses of the Honey Bee Brain to Infection with Deformed Wing Virus

**DOI:** 10.3390/v13020287

**Published:** 2021-02-12

**Authors:** Marie C. Pizzorno, Kenneth Field, Amanda L. Kobokovich, Phillip L. Martin, Riju A. Gupta, Renata Mammone, David Rovnyak, Elizabeth A. Capaldi

**Affiliations:** 1 Department of Biology, Bucknell University, Lewisburg, PA 17837, USA; ken.field@bucknell.edu (K.F.); amanda.kobokovich@bucknell.edu (A.L.K.); elizabeth.capaldi@bucknell.edu (E.A.C.); 2 Program in Cell Biology/Biochemistry, Bucknell University, Lewisburg, PA 17837, USA; plm015@bucknell.edu; 3 Department of Chemistry, Bucknell University, Lewisburg, PA 17837, USA; rag034@bucknell.edu (R.A.G.); david.rovnyak@bucknell.edu (D.R.); 4 Program in Animal Behavior, Bucknell University, Lewisburg, PA 17837, USA; renata.mammone@bucknell.edu

**Keywords:** deformed wing virus, RNA virus, *Apis mellifera*, honey bee, brain, transcriptome, disease, behavior

## Abstract

Managed colonies of European honey bees (*Apis mellifera*) are under threat from *Varroa destructor* mite infestation and infection with viruses vectored by mites. In particular, deformed wing virus (DWV) is a common viral pathogen infecting honey bees worldwide that has been shown to induce behavioral changes including precocious foraging and reduced associative learning. We investigated how DWV infection of bees affects the transcriptomic response of the brain. The transcriptomes of individual brains were analyzed using RNA-Seq after experimental infection of newly emerged adult bees with DWV. Two analytical methods were used to identify differentially expressed genes from the ~15,000 genes in the *Apis mellifera* genome. The 269 genes that had increased expression in DWV infected brains included genes involved in innate immunity such as antimicrobial peptides (AMPs), Ago2, and Dicer. Single bee brain NMR metabolomics methodology was developed for this work and indicates that proline is strongly elevated in DWV infected brains, consistent with the increased presence of the AMPs abaecin and apidaecin. The 1361 genes with reduced expression levels includes genes involved in cellular communication including G-protein coupled, tyrosine kinase, and ion-channel regulated signaling pathways. The number and function of the downregulated genes suggest that DWV has a major impact on neuron signaling that could explain DWV related behavioral changes.

## 1. Introduction

Viruses are a major threat to the health of managed honey bees (*Apis mellifera*) and have been implicated in colony losses and the syndrome referred to as colony collapse disorder [1,2,3]. The majority of viruses identified in honey bees are positive-sense single-stranded RNA viruses classified in two families (*Iflaviridae* and *Dicistroviridae*) in the order *Picornavirales* [4,5]. More recently, viruses with other types of genomes have been detected in bees [6], but little is known about their specific effect on honey bee physiology or behavior.

Deformed wing virus (DWV), a member of the *Iflaviridae* family [7], is one of the most common viruses detected in honey bees [8,9] and during an overt infection can produce adults with bloated and discolored abdomens and wing deformations [10]. The virus can be transmitted orally [11,12], sexually from infected drones to queens [13,14], vertically from infected queens to eggs [13,15], or vectored by the ectoparasitic *Varroa* mite [5,16]. DWV is responsible for over-wintering colony losses [17], and hives that are infested with the *Varroa* mite are more severely affected by DWV, have shorter life spans, and are more likely to collapse [18,19,20], suggesting that mite transmitted DWV results in a more severe disease state. This may be due to the route of entry, since oral infections are usually covert, do not reach the brain, and are overall less virulent [21,22]. When the virus is injected into developing pupa, the emerging adult bees often display deformed wings and the other classic symptoms of DWV infection [23], while injection of the virus into adult bees with fully formed wings does not produce these visible signs of infection. Even so, the virus can infect the head, replicate within the brain, and cause reduced life spans [20,21,24,25].

The functionality of a honey bee colony depends on a complex social structure including age polytheism, where the female workers complete tasks dependent upon their age and the needs of the colony [26]. There is growing evidence that DWV infection can affect the behavior of infected bees and alter the social structure and functionality of the colony [20,22,27]. Some studies have suggested that DWV causes bees to engage in behaviors related to foraging at an earlier age than uninfected bees [20,27], thereby altering the temporal polyethism of the hive. DWV may also affect the foraging abilities of infected bees by reducing the flight distance or time [20,28,29], thereby reducing their ability to contribute to colony homeostasis. Associative learning using the proboscis extension reflex in bees infected with DWV demonstrated reduced performance in learning acquisition and retention [22,30], and this reduction may be linked to the strain of DWV [22]. The closely related Kakugo virus (DWV-A) has been associated with aggressive behavior [31,32], though this was not confirmed in later studies using DWV [33]. Altogether, this suggests that infection with DWV, particularly via mite feeding, is a major cause of behavioral changes in honey bees that could destabilize polyethism, alter the function of workers, and lead to the collapse of the colony.

The goal of this experiment was to analyze the transcriptome of the honey bee brain after infection of adult bees with a high dose of deformed wing virus (DWV) to test the hypothesis that DWV infection changes the expression of genes involved in behavior and immunity. We analyzed the transcriptome of individual bee brains using RNA-Seq, rather than pooling samples, to provide a more robust analysis of the effect of DWV infection on the brain [34]. To confirm the RNA-Seq data, we developed a method to analyze the metabolome of individual bee brains. This work confirms a dramatic dysregulation of gene expression in the brain as a result of infection with DWV.

## 2. Materials and Methods

### 2.1. Honey Bees

Honey bee hives in the Bucknell University research apiaries were maintained using standard beekeeping techniques, but were not treated prophylactically against any disease. The colonies that were sources for all the bees in this experiment were queenright and contained a mixture of European subspecies, likely hybrids of *Apis mellifera mellifera*, *A. m. ligustica*, and *A. m. carnica*.

### 2.2. Virus Isolation and Infection of Honey Bees

Bees exhibiting deformed wings and presumed to be infected with DWV were collected from hives in the Bucknell apiary. A 2 g sample of bees was homogenized in 10 mL of phosphate buffered saline (137 mM NaCl, 2.7 mM KCl, 10 mM Na_2_HPO_4_, 1.8 mM KH_2_PO_4_, pH 7.4) using a tissue homogenizer and debris pelleted by centrifugation at 1500× *g* for 20 min at 4 °C. The resulting supernatant was filtered through a 0.2 μm filter and stored in aliquots at −80 °C for infections. RNA was isolated from a 400 μL aliquot of crude virus solution for pathogen screening and quantification of DWV genome equivalents according to Organtini et al. [35]. Using a standard curve of a plasmid containing the amplicon, the genome equivalents (GE) of viral RNA were calculated for the inoculum. Stocks of inoculum had minimum concentrations of DWV of 1 × 10^10^ GE/μL.

Frames of honeycomb containing capped pupae were collected from honeybee colonies with naturally mated queens and stored in a dark, humid incubator at 35 °C. Adult worker bees were removed from this comb 24 h post-eclosion, placed into wooden containers, and maintained in the incubator at 35 °C and 30% humidity. Bees were given ad libatum access to a slurry of 50% sugar syrup by weight mixed with pulverized pollen. Individual bees were anesthetized with carbon dioxide gas and injected into the side of the abdomen between the third and fourth sternites using a 10 µL Hamilton syringe with a 30 G needle. Mock infected bees (uninfected) were injected with 2 µL of sterile bee saline (55 mM NaCl, 35 mM KCl, 7 mM CaCl_2_, 20 mM MgCl_2_, 60 mM dextrose, 55 mM fructose, 15 mM sucrose) while DWV infected bees were injected with 2 µL of bee saline containing 10^8^ GE of DWV (1:200 dilution of original inoculum). Injected bees were held at 35 °C and 30% humidity for five days post-infection (dpi). Any bees that died before the end of the incubation period were removed from the experiment. At 5 dpi, bees were placed in individual vials and placed at 4 °C for 20 min to immobilize them. Using sterile forceps, the brains were dissected from the head capsule while floating in RNase-free bee saline and were immediately homogenized in Qiazol (QIAGEN, Germantown, MD, USA) using a sterile plastic pestle. Samples were stored at −80 °C until further processing.

### 2.3. RNA Isolation, Sequencing, and Data Analysis

RNA from individual brain samples was isolated using the Universal RNA Kit (QIAGEN) and quantified using a Nanodrop spectrophotometer (Thermo Scientific, Waltham, MA, USA). All RNA samples were subjected to RNA integrity analysis using a Bio-Analyzer (Agilent Technologies, Santa Clara, CA, USA). Due to the “hidden breakpoint” found in most insect 28S rRNA molecules [36], which causes the 28S rRNA to cleave under denaturing conditions and comigrate with the 18S rRNA, an RIN cannot be assigned. However, this analysis demonstrated that two of the samples had higher levels of RNA degradation and were removed from further analysis. Prior to RNA-Seq analysis, qRT-PCR analysis was used to quantify DWV RNA in each sample (data not shown). This analysis demonstrated that there was an average of 1 × 10^10^ GE/brain in the DWV infected bees, with the exception of one sample, which was removed from further analysis.

Library preparation from polyA selected RNA and RNA-Seq was carried out by the DNA Sequencing Center at Brigham Young University. Paired-end strand-specific reads of 125 nt in length were obtained using the Illumina HiSeq 2500 High-Output v4 PE 125 Cycle platform. Datasets were quality trimmed using Trimmomatic (v.0.35) with the parameters SLIDINGWINDOW:4:5 LEADING:5 TRAILING:5 MINLEN:25. Only reads with both pairs remaining after trimming were used for further analysis. The reads were mapped using STAR v.2.5.2a to the *Apis mellifera* genome, version 4.5 [37,38], containing ~15,000 annotated genes, with the DWV-A viral genome (PA strain, Genbank AY292384.1) included as a single transcript in the annotation so that all reads would map to the viral genome and the honey bee genome simultaneously. RSEM [39] was then used to predict the gene expression counts for each transcript according to the most current gene set available at the time (OGS 3.2). All sequence data files are publicly available in the NCBI Sequence Read Archive (SRA) under BioProject ID PRJNA691031.

To confirm that the bees were free of known honey bee viruses other than DWV-A, the reads were mapped using STAR v.2.5.2a simultaneously to the following viral genomes: acute bee paralysis virus (ABPV), NC_002548.1; bee macula-like virus, NC_027631.1; black queen cell virus (BQCV), KP119603.1; chronic bee paralysis virus (CBPV), NC_010711.1, NC_010712.1; DWV, AY292384.1; Israeli paralysis virus (IAPV), NC_009025.1; Kashmir bee virus (KBV), NC_004807.1; Lake Sinai virus (LSV), KM886905.1; Sacbrood virus (SBV), NC_002066.1; slow bee paralysis virus (SBPV), NC_014137.1; and Varroa destructor virus 1 (VDV 1/DWV-B), AY251269.2. None of the samples had reads mapping to the genome of any of these viruses other than the Pennsylvania strain of DWV-A (Genbank AY292384.1). The number of reads from some infected samples that mapped to the VDV-1/DWV-B were very low (less than 20) and represent less than 0.0001% of the total reads that mapped to DWV-A. This confirms that the bees were infected with a strain of DWV-A that closely matches the PA genome [7] and that the viral genome sequence did not drift significantly during the infection.

**Figure 1:**
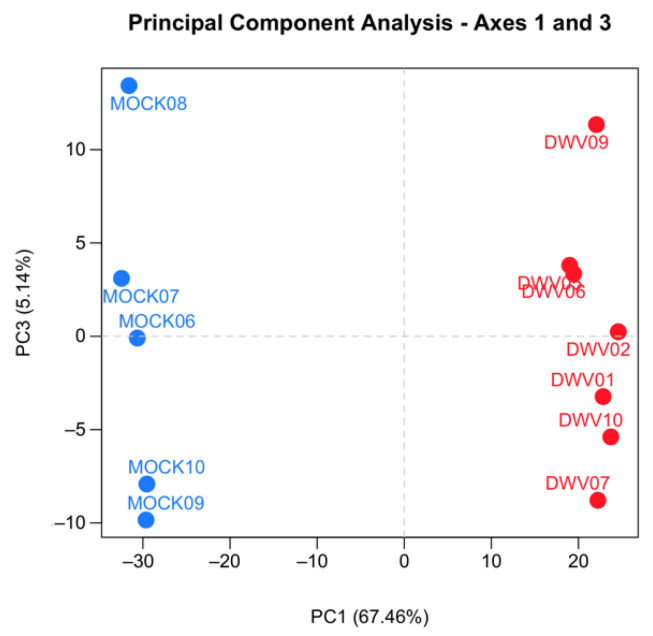
Principal component analysis (PCA) of the RNA-Seq data from the samples used in the final analysis. The gene expression profiles from the five mock (blue circles) and seven deformed wing virus (DWV, red circle) infected honey bees are presented. The PCA was performed on pairwise gene comparisons after variance-stabilizing transformation on the counts using DESeq2. Points that are closer together are more similar in gene expression patterns.

Samples were excluded from further analysis according to two predefined expectations. First, samples infected with DWV should show abundant expression of DWV transcripts, as demonstrated by both qRT-PCR results and the total number of reads mapping to the DWV-A genome, and mock-infected samples should show only background levels by both qRT-PCR and the number of DWV-A mapped reads. Two samples were excluded because they failed to meet these expectations. Second, mock-infected and infected samples were expected to cluster separately by hierarchical clustering after variance stabilizing transformation [40] and by principal component analysis. Four of the mock-infected samples co-clustered with the DWV-infected samples despite having no DWV transcripts (Figure S1). These samples were excluded from the mock-infected group and treated as their own group (MockB) for exploratory analysis. The read counts for all remaining samples were checked for quality control using SARTools v.1.6.1 [41]. We found that all samples had a similar proportion of genes with null read counts, density distributions of read counts, and expression of several “reference” genes. We also confirmed that samples within each treatment group were more similar to each other than to other samples by measuring the SERE statistic on pairwise scatter plots [42], hierarchical clustering after variance-stabilizing transformation, and principal component analysis (Figure S1 and Figure 1). These quality control analyses confirmed that the samples were comparable for differential expression analysis.

To confirm that the final experimental design still had sufficient statistical power, we performed Scotty analysis [43], using the gene expression results obtained from the current study. We used the following criteria to test the experimental design: two to 10 biological replicates per group; 10 to 100 million reads per sample; reads at an 80% alignment rate; detect at least 50% of expressed genes that are differentially expressed by a 2× fold change at *p* < 0.05; limit measurement bias by measuring at least 50% of genes with at least 50% of maximum power; costs of $174 per sample for library prep and quality control, $10 per million reads, and budget of $5000. Using these parameters and the gene expression data from this study, we found that a design of five replicates at a read depth of 10 million reads per sample, approximately 80% of the genes differentially expressed were detected at a 2-fold cutoff and an false discovery rate (FDR) of 0.05. This was well above our acceptable threshold of 50% of the differentially expressed genes. We therefore conclude that our study, which included at least five replicates for each treatment and an average read depth of 10 million reads had sufficient statistical power to detect differential gene expression.

SARTools v.1.6.1 [41] was used to analyze differential gene expression between the samples with edgeR v. 3.16.5 [44] or DESeq2 v.1.14.1 [45] after TMM-normalization and filtering transcripts without at least 1 TPM in half of the samples. We initially compared the results of edgeR and DESeq2 to determine if either differential expression method produced more reliable results for this dataset. We found very similar results from both analyses and we did not have an a priori reason to prefer one over the other, so we used the combined subset of differentially expressed genes identified by both methods for subsequent analysis. A FDR cutoff of 0.05 was initially used [46] to classify transcripts as differentially expressed and this threshold was lowered to 0.001 after it was found that thousands of transcripts were differentially expressed.

Differentially expressed genes that were identified by both DESeq2 and edgeR and had a fold change of 2.0 or greater were selected for functional analysis. Gene ontology analysis was performed on this list using g:Profiler version e95_eg42_p13_f6e58b9, database updated on 22/04/2019. Biological process functional categories were identified that had an adjusted *p*-value (g:OSC method) of 0.05 or less [47].

### 2.4. Nuclear Magnetic Resonance (NMR) Metabolomics

Individual bee brains, and their corresponding bodies, were obtained by manual dissection by one operator (EAC) in the presence of sterile PBS buffer and stored at −80 °C prior to use. To isolate aqueous metabolites, each brain or body was placed in a microcentrifuge tube and homogenized using bead-beating. A modified protocol by Bligh and Dyer (1959) was used and employed a chloroform and methanol solution in a 1:2 *v*/*v* ratio, referred to as solution A. A total of 0.5 mL of solution A and two small steel beads were added to a single bee brain in a microcentrifuge tube. The sample was homogenized for 60 s, and then 0.5 mL of chloroform added, followed by vortex agitation. Next, 0.5 mL of dd-H_2_O was added, followed by vortex agitation, and then centrifugation at 4 °C (2000 RCF) for 2 min. Authentic lower phase, a cleaning solution that will be referred to as solution B, was prepared by repeating the steps above in a separation funnel using the pure solvents and isolating the lower phase. The upper layer of the homogenized bee brain sample following centrifugation was transferred to a new microcentrifuge tube, to which 0.5 mL of solution B was added and the above steps repeated, which served to further wash and purify the aqueous metabolites. Following this wash step, the upper layer was transferred to a fresh tube and subjected to vacuum centrifugation for 4 h to remove solvent. For bee bodies, one large and one small steel bead were used for homogenization, which was extended to 3 min, with all other steps above being the same. Samples were resuspended in 0.5 mL of NMR buffer (99% D2O, 0.1 mM DSS, 100 mM phosphate, pH 7.4) and transferred to glass NMR tubes. One dimensional presaturation-NOESY 1H NMR spectra were acquired on a 14.1 T spectrometer (600 MHz for 1H, Varian Inc., Palo Alto, CA, USA, DDR1 generation console, vnmrj 3.2) using an inverse probe. All brain spectra utilized 512 transients and 32 steady-state transients, 4 s receiver time, a saturation period of 2 s, a NOESY mixing time of 160 μs, and a recycling delay of 9 s. All spectra of bodies used the same parameters except they were acquired with 64 transients. Spectra were profiled by a single rater (PLM) with the Chenomx NMR Suite 8.1 (Chenomx, Edmonton, AB, Canada) and are reported as concentrations (sample volume 0.5 mL).

## 3. Results

### 3.1. Analysis of Transcriptome of Infected Brains

**Figure 2:**
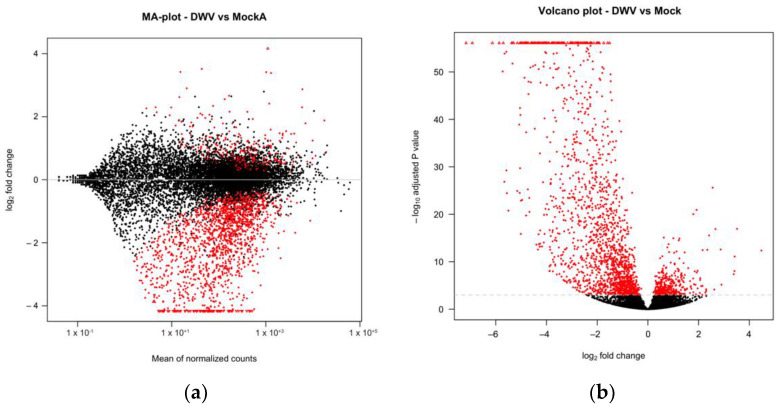
Expression of *A. mellifera* genes was compared by DESeq2 with MA (**a**) or volcano (**b**) plots. The mean expression level (log2 counts per million (CPM)), the fold change (log2 FC), and the FDR-adjusted *p* values are shown for each gene. Red points indicate differential expression (FDR ≤ 0.001 determined by DESeq2).

**Figure 3:**
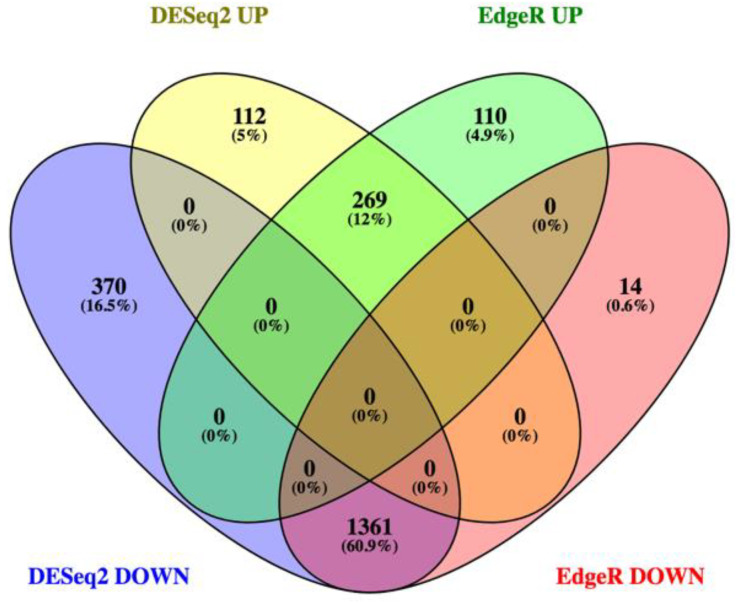
Venn diagram comparing the numbers of genes expressed at higher levels (UP) or lower levels (DOWN) identified by both DESeq2 and edgeR. The number of upregulated genes identified by both dESeq2 and edgeR was 269 while the number of downregulated genes was 1361. No gene was identified as upregulated by one algorithm and downregulated by the other.

We conducted transcriptomic analysis on RNA isolated from dissected bee brains that were either injected with DWV or mock infected with saline (Table 1). RNA-Seq results from individual samples were eliminated from further analysis following the criteria described in the Materials and Methods Section (Figure S1) and the five remaining mock and seven DWV infected samples were analyzed for differentially expressed genes. Using Scotty [43], we determined that the final study design, with a minimum of five replicates per group, was sufficiently statistically powerful to detect at least 50% of expressed genes that were differentially expressed by a 2× fold change at *p* < 0.05 (Figure S2). In addition, RNA reads were also mapped to the genomes of known honey bee viruses to identify unintentional co-infections that may confound data interpretation. The only known honey bee virus present in these samples was DWV-A, as less than 20 of the reads mapped to the DWV-B genome (VDV-1, AY251269.2) or other known viruses. The mock samples were compared to the DWV infected samples using principal component analysis, which shows that infection with DWV described over 60% of the observed variance in the count data (Figure 1). Differentially expressed genes (DEGs) were then analyzed using both the DESeq2 and edgeR programs [41] and a comparison of the DEGs expressed at either higher or lower levels in the DWV infected brains was shown using MA and Volcano plots (Figure 2). The imbalance between the DEGs was quite obvious with a greater number of genes were expressed at lower levels in the DWV infected brains. As the adjusted *p*-values associated with these DEGs were extremely low, only those up- or downregulated genes with adjusted *p*-values ≤ 0.001 were considered for further analysis. Even with this cutoff, the number of downregulated genes was over 1000 for both the DESeq2 and edgeR analyses. The two programs both identified 269 genes as upregulated in the DWV infected samples and 1361 genes downregulated compared to the uninfected samples (Figure 3, Tables S1–S4). There was more agreement between the two algorithms in identifying downregulated genes with edgeR only identifying an additional 14 downregulated genes, while DESeq2 identified an additional 370 downregulated genes. For the upregulated genes, each algorithm identified additional genes that were not identified by the other program. There were no genes that were identified as upregulated by one algorithm and downregulated by the other, or vice versa (Figure 3).

The copy number of DWV RNA in each sample was measured using qRT-PCR and in the DWV injected bees was found to be on average 10^10^ GE/brain (data not shown), which is similar to that seen in natural overt infections with DWV [28]. The number of reads mapping to the DWV genome comprised 20–40% of the total reads (Figure S3), demonstrating that the level of viral replication in the brain is high [24]. The level of DWV RNA measured in mock infected bees was over 10,000-fold lower and near the limit of detection using qRT-PCR (data not shown), which was confirmed by the low number of raw reads (less than 350) that mapped to the DWV-A genome in the mock infected samples (Table 1). This level of mapping (<0.0001%) likely represents endogenous reads mismapped to the DWV-A genome. The most abundantly expressed genes in the mock infected brains were GB40866 (heat shock cognate 4, a stress induced protein), GB53576 (apisimin, a component of royal jelly), and GB43247 (alpha-glucosidase, a salivary gland protein) (Figure S3). When the DWV reads were removed from the analysis, the most abundant genes expressed in the DWV infected samples included GB50196 (long wavelength opsin, a protein expressed in the eye), GB47977 (titin, a component of muscle) as well as the same heat shock protein expressed in the mock samples. The variability of these highly expressed genes between samples suggests contamination with other tissues during dissection of the honey bee brains. Muscles, salivary glands, and other connective tissues surround the brain in the bee’s head capsule; the visual pigments associated with the compound eye are also directly connected to brain tissues. As a result, it is possible that some of those tissues were present on the brain prior to RNA isolation. However, the brain dissections were performed blind to the treatment group and therefore any possible contamination would be similar across the samples, which was supported by analysis of the RNA-Seq data.

**Figure 4:**
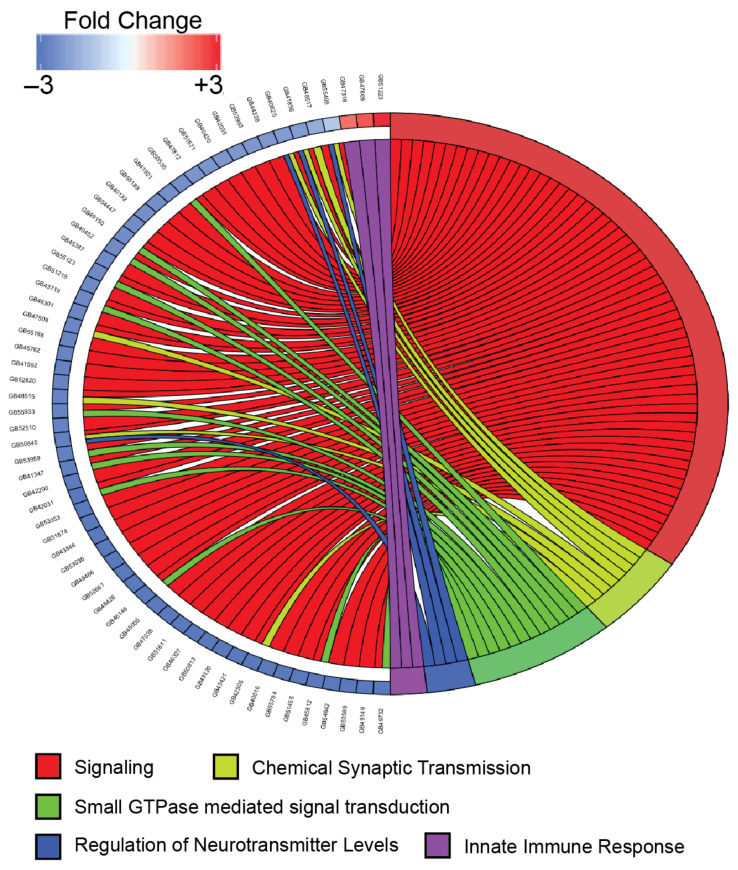
Chord plot comparing the expression levels of selected genes and their relationship to specific biological process gene ontology terms. Gene expression changes are shown for selected genes that were differentially expressed (FDR < 0.001 by DESeq2 and edgeR) and represented in the indicated gene ontology category. Expression level changes (log2 fold change) are shown for the comparison of DWV-infected samples to mock-infected samples. Connections from the right side of the figure to the left signify associations between genes and selected biological process categories. Genes are shown in the following biological process categories: GO:0023052 (signaling (red)), GO:0007268 (chemical synaptic transmission (light green)), GO:0007264 (small GTPase mediated signal transduction(green)), GO:0001505 (regulation of neurotransmitter levels (blue)), and GO:0045087 (innate immune response(purple)).

Gene ontology analysis identified biological processes enriched in the upregulated genes related to defense and immunity (Table 2, Figure 4), though only four specific genes including those encoding the AMPs hymenoptaecin and abaecin were overrepresented in these categories. Previously published genes expressing proteins involved in immunity and defense [48,49] highlight additional DEGs in both the upregulated and downregulated lists that are involved in immunity (Table 3 and Table 4). Gene ontology analysis of the downregulated genes showed an enrichment in over 80 biological processes (Table 2), demonstrating the diversity of cellular pathways affected by DWV in the brain including those involved in multiple signal transduction pathways and synapse function (Figure 4).

### 3.2. Analysis of Metabolome of Infected Brains

**Figure 5:**
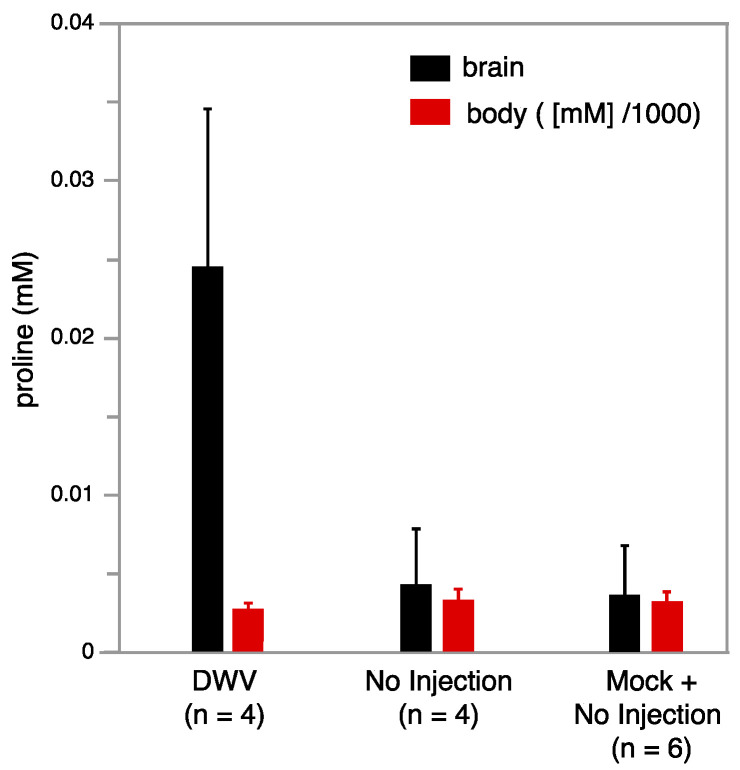
Average proline concentration in 0.5 mL NMR buffer as a function of DWV state for bee brains (left hand black bars) and corresponding bodies (right hand red bars) is illustrated. Proline increases strongly in the brains of DWV infected workers, but is relatively unchanged in corresponding bodies. The proline concentration in 0.5 mL NMR buffer extracted from the corresponding bodies was much higher and its value was scaled down by 1000 (e.g., for the bodies the scale corresponds to M).

Metabolomics studies were conducted using NMR on individual brain tissue from bees that were not injected (no injection), injected with saline (mock), or injected with DWV. The use of an inverse probe (proton optimized), extended acquisitions (2 h for each bee brain sample), and relatively high field strengths (14.1 T) resulted in the ability to assign and quantify 25 aqueous metabolites in a single bee brain (Tables S7 and S8). Four worker bees with injected DWV, four that did not receive injections, and two additional workers who received mock injections were studied. These ten samples were dissected to obtain the brain and the corresponding bodies were retained separately, which were subsequently homogenized and extracted via a chloroform-methanol procedure to yield NMR-quality samples. Notably, a particularly strong trend was observed with free proline, which was strongly upregulated in the brains of the DWV infected workers, whereas the levels in workers who received no injection or a sham injection were lower by almost an order of magnitude (Figure 5). In contrast, the proline levels were not significantly changed in the DWV injected worker bodies, indicating that this metabolic response is localized to the brain. While the brain proline change was particularly strong, other metabolites of interest are suggested by this work (Tables S7 and S8) and further study on larger samples will be needed to determine their significance.

## 4. Discussion

Since the publication of the honey bee genome [38], numerous studies have analyzed the *Apis mellifera* transcriptome under a variety of conditions including natural or induced infection with one of the many pathogens that plague bees [50,51,52,53,54]. While most of these studies analyzed whole bees or body segments (thorax and/or abdomens) and isolated RNA from pooled samples, we chose to analyze DEGs in individual bee brains, which has been done only in a few studies [55,56]. Isolated tissue, even though it may be composed of different cell types, is a more representative way to study gene expression changes [34]. Injection of DWV into the abdomen simulates infection by the *Varroa* mite and results in pathogenic infection of the brain [21,24] and while the dose injected for this study was high, it is within the range that could be transmitted by a single mite during feeding [57]. Under these conditions, a number of genes are upregulated by infection while an even higher number are downregulated, pointing to the dramatic effect that DWV has on the function of the bee brain.

A primary hypothesis of this work was that expression of genes involved in immunity would be upregulated in virus infected brains. This hypothesis is supported by the gene ontology analysis in which biological processes related to defense and immunity were enriched in the upregulated gene set (Table 2, Figure 4). Comparing the DEGs in this study to a broad list of honey bee genes involved in immunity [48,58] or a more specific suite of genes shown to be upregulated in other transcriptome studies after infection of a pathogen [49] showed that additional immunity genes were upregulated in DWV infected brains (Table 3). These include antimicrobial peptides (AMPs; abaecin, apidaecin, and hymenoptaecin), components of the RNAi pathway (Dicer, AGO2, maelstrom, and PRM1), which is the primary antiviral defense system in insects [59] and multiple members of the Toll pathway (cactin, NFkB p100, and PGPR-S2). AMPs were originally described as proteins upregulated during bacterial infection [60], but are also known to be activated by viral infection [52]. The increased expression of several AMPs in DWV infected brains, in contrast to orally infected pupae [52], demonstrates that the brain is mounting an immune response to the virus, even if it is not robust enough to inhibit viral replication. However, the number of immunity genes induced by infection appears low compared to other studies [48,49] and suggests that the brain is not capable of mounting as robust a defense against DWV. In addition, there is also a number of immunity genes repressed during virus infection (Table 4), and while this may be the result of a global reduction in gene expression (discussed below), it is supported by previous work showing that DWV inhibits the expression of immunity genes [61,62] and may be able to inhibit cellular immunity, particularly the Jak/Stat pathway and Toll-like receptors. Together, these possibilities may explain how DWV is capable of replicating to such high levels in the brain; in some of the infected samples, 30–40% of the RNA reads mapped to the DWV genome (Figure S3). In addition, some of the genes overexpressed during infection are host proteins that might be required for viral replication. While the biological processes enriched in upregulated genes do not point to a particular cellular pathway that the virus targets, this study provides candidate genes that could be studied in the future to identify cellular proteins involved in viral replication.

The expression of the AMPs is supported by metabolomic data (Figure 5), which demonstrates a significant increase in free proline in the brain after infection and could be related to the overexpression of abaecin and apidaecin, which are composed of nearly 20% prolin [63,64]. The study of aqueous metabolites using NMR offers high reproducibility, accurate metabolite identification, and precision of quantitation [65]. While it has been used to characterize relationships between disease states and biochemical pathways in humans, it is less commonly applied to insects [66]. Specialized instrumentation for the study of single insects has been developed [66], but analysis of single insects creates limitations of sensitivity for NMR, and interrogating the chemical composition of isolated tissues incurs a further loss of sensitivity. The NMR methodology developed here (see Materials and Methods) was used to measure aqueous metabolites of isolated bee brains and supports the differences observed in the gene expression changes.

The total number of downregulated genes during infection and their level of repression is surprising and suggests that DWV may be inducing a general inhibition of cellular gene expression. Polio, a distant relative of DWV [7], is known to dramatically inhibit cellular transcription and translation [67] by a variety of host-shut off mechanisms in order to direct cellular resources to viral synthesis. However, since the majority of genes in our data were not differentially expressed and many were upregulated (Figure 2a,b), infection with DWV does not appear to induce a global inhibition of cellular gene expression suggesting that DWV is not cytotoxic. This is consistent with work showing that DWV may not be as virulent as other honey bee viruses [52,68,69], though this may depend on the genotype of DWV or the mode of transmission [21], and is supported by observations that the honey bee AmE-711 cell line is persistently infected with DWV [69] and that colonies can maintain low levels of virus infection in the absence of mites [70]. Together, DWV does not appear to be highly virulent to neurons and may affect behavior without actually leading to cell or organismal death.

From the analysis of biological processes enriched in the set of downregulated genes, it is clear that transcripts encoding proteins involved in cell signaling, cell communication, and synaptic function are dramatically lower in DWV infected brains (Table 2), suggesting that DWV replication inhibits neuronal physiology and brain function. Multiple signaling pathways are affected including G-protein coupled receptors (GPCRs), tyrosine kinase receptors (TKs), and ion-channels. The GPCR category includes examples of receptors that respond to biogenic amines and have roles in a wide range of behaviors such as learning, memory, and foraging [71,72,73], and are distributed in regions of the brain where DWV replicates [72,73]. Other GPCRs that are repressed include members of the dopamine, SIFamide, and Neuropeptide Y receptor families, which have also been linked to various behaviors [74,75,76,77]. The other biological processes involved in neuronal function such as synaptic signaling, small GTPase mediated transduction, and ion transport contain additional genes whose decreased expression could explain the behavioral changes seen in DWV infected bees and which provide candidate genes for further in-depth exploration. One explanation for the repression of so many genes involved in signaling is that levels of transcription factors shown to be master regulators of various “neurogenomic states” [78] are affected by the virus. Even though biological processes related to transcriptional regulation were not enriched, several of these transcription factors were found in the DEGs reported here including forkhead box proteins (fox), ftz transcription factor 1 (ftz-f1), broad complex (br), dorsal (dl), and delta (Dl), which are all expressed at lower levels, and fruitless (fru), which is more abundant in DWV infected brains. Any of these could be involved in regulating the transcription of the DEGs, leading to reduced neuron signaling, altered brain function, and aberrant behavior. In situ hybridization experiments have previously shown that DWV viral RNA and replication is located in critical regions of the brain [24,32] and have been shown to be involved in vision, olfaction, and integration of sensory stimuli [79]. If virus replication within neurons is downregulating the expression of important signaling pathways and inhibiting the function in those brain regions, it is reasonable to expect that a bee’s ability to respond to external stimuli or process signals would be affected.

In conclusion, transcriptomic analysis of brain tissue from bees instrumentally infected with DWV showed increased expression of a limited set of genes involved in immune response and a massive and extensive decreased expression of genes involved in multiple signaling pathways, suggesting a systemic effect on brain function. These changes in gene expression match well with behavioral studies describing decreases in learning and memory in DWV infected bees [22,30] and suggest that adult bees infected with DWV can remain alive but suffer significant deficits in behavioral pathways required for performing their normal duties and contributing to the work of the colony. While it is likely that gene expression profiles will vary depending on individual colony differences, this work provides candidate genes for future studies on DWV infection of the brain.

## Figures and Tables

**Table 1:** Information about the analyzed RNA-Seq samples.

Sample	Treatment ^1^	Analysis Group	Reads Mapping to DWV-A Genome ^2^	Input Reads	Mapped Reads	% of Reads Mapped
Mock06	saline	Mock	184	9,777,028	8,780,845	89.8%
Mock07	saline	Mock	166	9,941,632	8,974,412	90.3%
Mock08	saline	Mock	333	10,056,939	8,902,211	88.5%
Mock09	saline	Mock	193	12,158,954	10,995,539	90.4%
Mock10	saline	Mock	257	14,885,921	13,471,208	90.5%
DWV01	DWV 10^8^ GE	DWV	372,674	9,381,298	8,317,765	88.7%
DWV02	DWV 10^8^ GE	DWV	536,845	12,014,963	10,737,245	89.4%
DWV05	DWV 10^8^ GE	DWV	627,575	11,463,725	10,403,578	90.8%
DWV06	DWV 10^8^ GE	DWV	600,523	12,942,641	11,667,059	90.1%
DWV07	DWV 10^8^ GE	DWV	282,418	10,940,452	9,708,615	88.7%
DWV09	DWV 10^8^ GE	DWV	284,126	13,132,427	10,299,986	78.4%
DWV10	DWV 10^8^ GE	DWV	1,047,479	19,577,082	17,478,913	89.3%

^1^ Inoculum injected into the abdomen of one-day old bee. ^2^ Raw number of reads mapped to the DWV-A genome (AY292384.1).

**Table 2:** Selected biological processes enriched in upregulated or downregulated differentially expressed genes (DEGs).

DEG Class	GO: Category	GO: Biological Process Term	*p* ^1^	#DEG ^2^	#Category ^3^
Upregulated	0045087	Innate immune response	1.04 × 10^−4^	3	12
	0006955	Immune response	4.56 × 10^−4^	3	19
	0006950	Response to stress	1.99 × 10^−2^	4	188
Downregulated	0007154	Cell communication	2.03 × 10^−28^	152	775
	0007165	Signal transduction	1.50 × 10^−26^	140	752
	0007186	G-protein-coupled receptor signaling pathway	3.74 × 10^−9^	30	136
	0007166	Cell surface receptor signaling pathway	2.32 × 10^−8^	32	129
	0007264	Small GTPase mediated signal transduction	9.57 × 10^−6^	20	71
	0006811	Ion transport	6.64 × 10^−5^	307	49
	0018108	Peptidyl-tyrosine-phosphorylation	1.86 × 10^−4^	11	32
	0007169	Transmembrane receptor protein tyrosine kinase signaling pathway	3.20 × 10^−3^	7	14
	0099536	Synaptic signaling	2.97 × 10^−3^	11	32
	0051056	Regulation of small GTPase mediated signal transduction	5.03 × 10^−3^	12	42

^1^ Overrepresented *p*-value for this category. ^2^ Number of differentially expressed genes in this category identified by g:Profiler with a *p* ≤ 0.05 cutoff. ^3^ Total number of genes in this biological process category.

**Table 3:** Upregulated immunity genes.

OGS 3.2 ID	Pathway ^1^	Gene Symbol	Gene Description	List ^2^
GB45495	Heat shock proteins	LOC411700	heat shock protein 83-like	E
GB49918	IMD	LOC724728	NF-kappa-B essential modulator	D
GB41606	JNK	LOC726947	TGF-beta-activated kinase 1 and MAP3K7-binding protein 3	E
GB52625	MAPK	pnt	ETS-like protein pointed	D
GB48923	RNAi	LOC726766	endoribonuclease Dicer	B
GB50955	RNAi	LOC411577	protein argonaute-2, AGO2	B
GB54480	RNAi	PRM1	TAR RNA-binding protein 2	E
GB54808	RNAi	LOC409557	protein maelstrom	B
GB52596	Serine proteases	nanos	protein nanos	D
GB43738	Toll	PPO	phenoloxidase subunit A3	B
GB46708	Toll	LOC552594	cactin	E
GB47805	Toll	Pgrp-s2	peptidoglycan recognition protein S2	B
GB46236	Toll, AMP	LOC100576979	apidaecin type 73-like	E
GB47318	Toll, AMP	LOC406144	abaecin	B
GB47546	Toll, AMP	Apid1	apidaecin 1	B
GB51223	Toll, AMP	LOC406142	hymenoptaecin	B
GB51306	Toll, AMP	LOC406115	apidaecin	B
GB40654	Toll, IMD	LOC552247	nuclear factor NF-kappa-B p100 subunit, relish	B
GB50013	Toll, PPO	LOC726126	proclotting enzyme, serine protease 8	E
GB42981	Toll/TLR	B-gluc2	beta-1,3-glucan recognition protein 2	E
GB52625	MAPK	pnt	ETS-like protein pointed	D
GB48923	RNAi	LOC726766	endoribonuclease Dicer	B
GB50955	RNAi	LOC411577	protein argonaute-2, AGO2	B

^1^ Pathway of each gene is a member of as described in [48,49]. ^2^ Algorithm that identified the gene; B = both, D = DESeq2 only, E = edgeR only.

**Table 4:** Selected downregulated immunity genes identified by both DESeq2 and edgeR.

OGS 3.2 ID	Pathway ^1^	Gene Symbol	Gene Description
GB52453	Apoptosis	LOC100578356	apoptotic protease-activating factor 1-like
GB56010, GB56012	Apoptosis, JNK	LOC409286	stress-activated protein kinase JNK
GB47938	C-lectin domain	CTL4	C-type lectin 4
GB51399	C-lectin domain	CTL8	C-type lectin 8
GB52628	Heat shock proteins	Hsf	heat shock factor
GB44117	IG Superfamily Genes	IGFn3-5	immunoglobulin-like and fibronectin type III domain containing 5
GB45752	Imd	Ubc13	ubiquitin-conjugating enzyme 13
GB48187, GB48188	imd	LOC413809	mitogen-activated protein kinase kinase kinase 7
GB42200	Jakstat	LOC408577	phosphatidylinositol 3-kinase regulatory subunit alpha
GB43421	Jakstat	LOC412008	sprouty-related, EVH1 domain-containing protein 1, Spred
GB47412, GB47413	Jakstat	LOC411982	E3 ubiquitin-protein ligase CBL
GB48204	Jakstat	LOC413980	CD109 antigen, TEPA
GB50020	both	LOC413742	signal transducer and activator of transcription 5B, STAT92E-like
GB52510	Jakstat	LOC413772	suppressor of cytokine signaling 7, Socs7
GB47812	PI3K-Akt-Tor	RPTOR	regulatory associated protein of MTOR, complex 1
GB40977	RNAi	LOC552259	staphylococcal nuclease domain-containing protein 1, Tudor-SN
GB42279	RNAi	LOC726768	ATP-dependent RNA helicase dbp2-like
GB48208	RNAi	LOC552062	protein argonaute-2, AGO1
GB50259	RNAi	LOC410580	synaptic functional regulator FMR1
GB44031	Toll	Dl	dorsal
GB48426	Toll	LOC410235	toll-like receptor Tollo, Toll-10
GB43456	Toll/TLR	18-w	18-wheeler
GB52453	Apoptosis	LOC100578356	apoptotic protease-activating factor 1-like
GB56010, GB56012	Apoptosis, JNK	LOC409286	stress-activated protein kinase JNK

^1^ Pathway of each gene is a member of as described in [48,49].

## Data Availability

The RNA-Seq data will be made publicly available via the NCBI Sequence Read Archive.

## Supplementary Materials

The following are available online at https://www.mdpi.com/1999-4915/13/2/287/s1, Figure S1: Principal component analysis of all mock and DWV infected samples, Figure S2: Power analysis of differential gene expression, Figure S3: Most highly expressed genes in each sample as analyzed by DESeq2, Table S1: Complete list of all upregulated genes identified by DESeq2, Table S2: Complete list of all upregulated genes identified by edgeR, Table S3: Complete list of all downregulated genes as identified by DESeq2, Table S4: Complete list of all downregulated genes identified by edgeR, Table S5: Complete list of Gene Ontology terms and statistical information of upregulated genes, Table S6: Complete list of Gene Ontology terms and statistical information of downregulated genes, Table S7: Average aqueous metabolite concentrations (nM) in worker bee brains, Table S8: Average aqueous metabolite concentrations (nM) in corresponding bee bodies.
